# Thrombus in Transit: Extract or Dissolve?

**DOI:** 10.7759/cureus.9550

**Published:** 2020-08-04

**Authors:** Vishal R Dhulipala, Bolajoko O Fayoda, Htoo Kyaw, Cesar Ayala-Rodriguez

**Affiliations:** 1 Internal Medicine, The Brooklyn Hospital Center, Brooklyn, USA; 2 Cardiology, The Mount Sinai Hospital, New York, USA; 3 Internal Medicine/Cardiology, The Brooklyn Hospital Center/Mount Sinai Heart, Brooklyn, USA; 4 Cardiology, The Brooklyn Hospital Center/Mount Sinai Heart, Brooklyn, USA

**Keywords:** thrombolytics, transthoracic echocardiography, pulmonary embolus, cerebrovascular accident, hemorrhagic transformation, patent foramen ovale, anticoagulation, embolectomy, right ventricle, deep vein thrombosis (dvt)

## Abstract

Thrombus in transit (TIT) remains an uncommon diagnosis. However, it is often found in patients diagnosed with acute pulmonary embolism (PE). While thrombolytics are mainly used in life-threatening presentations, their role in stable patients with a known history of intracranial hemorrhage (ICH) is unclear.

## Introduction

It is understood that thrombus in transit (TIT) has its origin via a deep vein. Moreover, the formation of a thrombus can be due to an intracardiac primary condition such as atrial fibrillation or heart failure. This can be complicated by a cerebrovascular accident (CVA) from an embolic stroke, in the presence of a cardiac defect such as patent foramen ovale (PFO). A thrombus can originate from a deep vein, particularly in the lower extremity, and is comprised of fibrin, erythrocytes, leukocytes, and platelets, which coagulate in an unorganized fashion. This case report focuses on the therapeutic approach in managing TIT in a patient with a known history of intracranial hemorrhage (ICH). The critical issue we faced was whether to extract or dissolve a right heart thrombus. Our approach to this complex case did not involve standard therapy. Of note, no trials or studies have yet described a clear pathway to the optimal management of this condition.

## Case presentation

**Figure 1:**
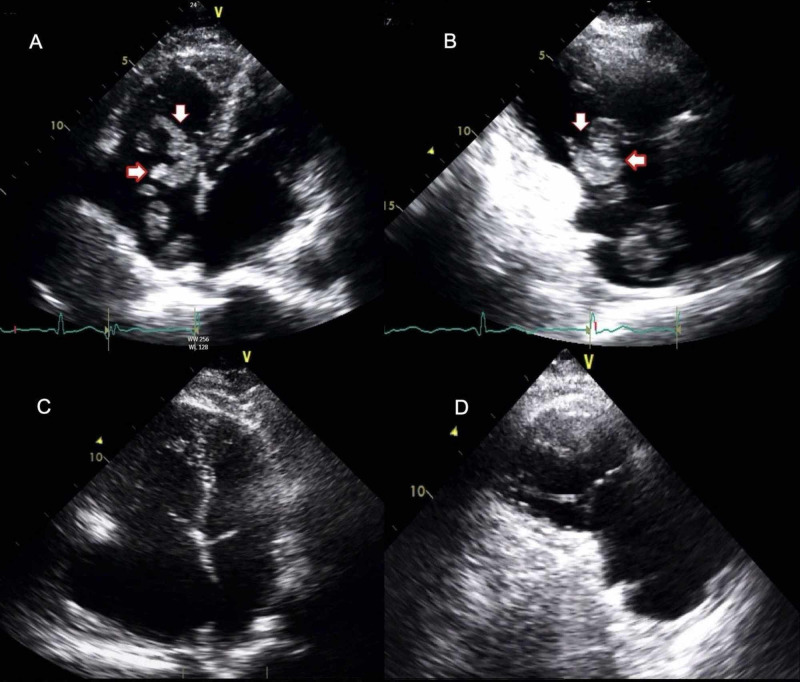
Transthoracic echocardiography of thrombus in transit Apical four-chamber view (A) and right ventricular inflow view (B) at admission, showing thrombus across the tricuspid valve (arrows). Apical four-chamber view (C) and right ventricular inflow view (D) after thrombolytic therapy showing the resolution of thrombus

A 63-year-old male with a history of atrial fibrillation on anticoagulation, ischemic cerebrovascular accident (CVA) with hemorrhagic transformation (HT), and craniotomy two years prior was admitted for shortness of breath. The patient was subsequently diagnosed with a sub-massive pulmonary embolism (PE) on computed tomography angiography (CTA) of the chest. Transthoracic echocardiography (TTE) performed with an apical four-chamber view (Figure 1A) demonstrated a 3.4 x 2.8-cm TIT. Figure 1B shows the right ventricle (RV) inflow view picturing a thrombus across the tricuspid valve with moderate RV dilation. The patient was hemodynamically stable and initially managed with an intravenous heparin drip. The activated partial thromboplastin time (aPTT) goal of 40-60 seconds was achieved. After a thorough evaluation, the team engaged in a medical discussion with the patient and his family. This ultimately led to a decision by the team to intervene with the use of localized thrombolytic therapy. Shortly after discussing the risks and benefits of the planned procedure, consent was obtained. Access was gained via the right internal jugular vein, and a 5 French catheter was introduced through superior vena cava (SVC) into the right atrium. At this juncture, alteplase was locally infused via the catheter at a rate of 2 mg per hour (0.05-0.1 mg/kg/hr) for eight hours. The thrombus previously identified in the right heart was no longer seen on a follow-up echocardiogram. Figure 1C shows the apical four-chamber view, and Figure 1D shows the RV inflow view with the total dissolution of the previous clot witnessed prior to intervention. The presenting symptom in our patient soon improved, and after several days, he was discharged home on anticoagulation with warfarin [international normalized ratio (INR): 2-3].

## Discussion

A thrombus in its nature will be in a transit form. However, its severity on clinical outcome is characterized after its embolization. A deep vein thrombus is a marker for venous thromboembolism (VTE); moreover, the location of that thrombus (iliac vs. femoral vs. popliteal veins) can be a cause of concern for PE and right heart thrombus. About 98% of TIT cases are known to be associated with PE [1]. There are therapeutic approaches available for the management of thrombi or emboli wherever it is located. A sub-massive PE accompanied by right heart thrombus put our patient at great risk of mortality without intervention, and existing literature does not provide a clear approach to standard management of the condition [2,3]. However, some studies have reported improved survival with thrombolytic therapy alone [4]. Right heart TIT is stated to have a mortality rate of >25%, and it occurs in about 4% of patients with PE [1,5]. Our patient’s TIT likely occurred due to a blood clot formation in the lower extremity and/or in combination with atrial fibrillation formation in situ [5].

The use of TTE is helpful and recommended in patients diagnosed with massive or submassive PE to identify the right heart thrombus [4-6]. Failure to promptly identify a TIT poses a threat to the patients' clinical outcome [5]. TIT is classified into various types based on its echogenic shape and appearances; type A thrombus is the most common one and occurs as a result of thrombosis of deep veins with a high risk of emboli. Otoupalova et al. have further described it as “elongated, with a worm-like appearance” and mobile in nature within the right heart chambers [5]. Type B thrombus originates within heart chambers (atrium or ventricle). This thrombus is described as “firmly attached” to the heart chamber wall and appears “in an ovoid shape” [5]. Type C thrombi are uncommon; they are described to have a high degree of mobility and share the characteristics of type A as well as type B right heart thrombi [5,6]. Our patient’s TTE showed a TIT across the tricuspid valve with moderate RV dilation (Figures 1A, 1B). The characteristics of our patient’s TIT indicated that it was of type A, with elongation, mobile in nature, and worm-like appearance. Our patient was hemodynamically stable, and this was supported by the fact that there was no identified evidence of RV dysfunction on TTE. Therefore, if the pulmonary circulation were obstructed due to an embolus, pulmonary arterial pressure would increase to levels that would result in a proportional change to the pulmonary circulation; this could cause right heart strain and RV dysfunction leading to a fatal outcome.

Give our patient’s known history of ischemic CVA with HT and craniotomy two years prior, it became a matter of concern due to our chosen method of management and treatment of the diagnosed TIT. Our patient did not have any cardiac defects such as PFO, which could trigger an embolus. However, the known history of atrial fibrillation remained a risk for a right heart thrombus, cardio-embolic cerebral infarction, and severity of hemorrhagic transformation with worsening clinical stroke outcomes [7]. A Head CT scan or MRI is indicated in patients with cerebral infarction. Hypo-attenuating foci are often identified in the affected region and edema, creating the loss of gray matter on imaging. Some studies have reported the incidence of HT of up to 20% [7]. It is clear that cerebral infarction is a major risk factor associated with HT. In our case, the patient had a history of CVA complicated by HT. A prospective study that looked at 1,495 patients with previous ICH receiving thrombolytic therapy has shown no significant increase in the risk of HT [8]. It was our priority to establish this fact since our treatment modality relied on the use of anticoagulation and/or thrombolytics. While thrombolytics are mainly used in life-threatening presentations, their role in stable patients with a known history of ICH is still unclear.

There are multiple reports of high mortality rates in patients who are diagnosed with TIT with PE pathology [2,3,5,9]. The optimal method for the management of this patient population remains unclear and clinical data to support any kind of intervention is scarce. However, current treatment modality revolves around anticoagulation, thrombolytics (systemic versus percutaneous catheter-directed thrombolysis with or without the use of ultrasound-enhancement), and surgery (thrombectomy or embolectomy). The use of thrombolytics in patients with PE and unstable hemodynamics are well described in a metanalysis that looked at pooled reports between 1992 and 2013 [9]. This report involved an analysis of the comparative efficacy of different treatment modalities in 328 patients with right heart thrombi and diagnosed PE. The study favored thrombolytics and/or surgery. Nonetheless, the estimated probability of survival in hemodynamically stable and unstable patients was better with thrombolysis (92.4% and 81.5%, respectively) than with surgery (86.9% and 70.4%, respectively) or anticoagulation (71.8% and 47.7%, respectively) [9].

In another retrospective analysis by Rose et al. in 2002, 177 cases of right heart thromboembolism were divided into four categories based on treatment as no therapy (9%), anticoagulation therapy (35.0%), surgical procedure (35.6%), and thrombolytic therapy (19.8%) [4]. The mortality rate associated with no therapy, anticoagulation therapy, surgical embolectomy, and thrombolysis was 100.0%, 28.6%, 23.8%, and 11.3%, respectively. This shows more effective therapy as well as a better outcome with the use of thrombolytics versus either anticoagulation alone or aggressive surgical intervention [4]. The challenge we faced in our case pertained to the question as to what appropriate method would yield the best outcome despite the patient's known history of CVA with HT and craniotomy two years prior. The method to treat a thrombus or embolus can vary based on the patient's presentation and history, and it can affect the severity of a patient’s condition, raising the stakes for a successful outcome. Exercising the use of thrombolysis in a percutaneous approach yielded a direct pathway for dissolving a clot in the right atrial heart chamber without complications in this patient. Hence, dissolving a TIT should be seriously considered as it leads to more effective management with a better outcome over surgical extraction or anticoagulation.

The initial use of anticoagulation with heparin infusion in our patient was deferred due to its documented high failure rate. According to a metanalysis, about 40% of patients started on anticoagulation had to be shifted to either treatment with thrombolytics or surgical approach due to clinical deterioration [9]. Although there was no evidence of worsening symptoms or hemodynamics in our patient, concern for a potential worsening clinical outcome prompted us to choose percutaneous catheter-directed thrombolysis therapy. Despite the lack of data in the current literature, the infusion of alteplase at a rate of 2 mg per hour (0.05-0.1 mg/kg/hr), a low therapy dose, for a period of eight hours proved a success as shown on repeat TTE. Another case series have reported thrombus actively dissolving to 50% within two hours of initiating catheter-directed thrombolysis, culminating in total dissolution in 24 hours [10].

In our case, TTE revealed an echogenic mass with elongation, mobility, and worm-like appearance across the tricuspid valve, which was consistent with type A thrombus and RV dilation with mildly depressed RV function. A sub-massive PE put our patient at great risk of mortality without further intervention. While there was no complication in the outcome for our patient, concern regarding the prior history of ICH can often lead to the exclusion of thrombolysis from the management of this condition, thereby provoking re-bleeding or HT.

## Conclusions

We face several challenges in the management of right heart thrombus with PE pathology, and one of the many shortcomings is the scarcity of data on the optimal therapeutic approach. Anticoagulation, thrombolytic (systemic or catheter-directed), or surgical options can be considered for the management of right heart thrombus. In this case, the presence of comorbidities and periprocedural complications prompted us to opt for catheter-directed thrombolytic infusion in the patient. This case highlights the role of catheter-directed thrombolytic treatment modality in a high-risk case with a concern for bleeding complications. However, the role of thrombolytics in patients with prior history of ICH who presents with right atrial thrombus and sub-massive PE remains unclear. Based on current evidence, dissolving the right heart clot appears to be the best approach, and it is highly favored over surgical extraction with some documented proof of better outcomes. However, optimal dosing and treatment duration of thrombolytic therapy should be defined in a large randomized study to validate its therapeutic risks and benefits.
